# A Computationally Efficient, Exploratory Approach to Brain Connectivity Incorporating False Discovery Rate Control, *A Priori* Knowledge, and Group Inference

**DOI:** 10.1155/2012/967380

**Published:** 2012-11-04

**Authors:** Aiping Liu, Junning Li, Z. Jane Wang, Martin J. McKeown

**Affiliations:** ^1^Department of Electrical and Computer Engineering, University of British Columbia, Vancouver, BC, Canada V6T 1Z4; ^2^Laboratory of Neuro Imaging, Department of Neurology, UCLA School of Medicine, Los Angeles, CA 90095, USA; ^3^Division of Neurology, Department of Medicine and Pacific Parkinson's Research Centre, University of British Columbia, Vancouver, BC, Canada V5Z 1M9

## Abstract

Graphical models appear well suited for inferring brain connectivity from fMRI data, as they can distinguish between direct and indirect brain connectivity. Nevertheless, biological interpretation requires not only that the multivariate time series are adequately modeled, but also that there is accurate error-control of the inferred edges. The PC_fdr_ algorithm, which was developed by Li and Wang, was to provide a computationally efficient means to control the false discovery rate (FDR) of computed edges asymptotically. The original PC_fdr_ algorithm was unable to accommodate *a priori* information about connectivity and was designed to infer connectivity from a single subject rather than a group of subjects. Here we extend the original PC_fdr_ algorithm and propose a multisubject, error-rate-controlled brain connectivity modeling approach that allows incorporation of prior knowledge of connectivity. In simulations, we show that the two proposed extensions can still control the FDR around or below a specified threshold. When the proposed approach is applied to fMRI data in a Parkinson's disease study, we find robust group evidence of the disease-related changes, the compensatory changes, and the normalizing effect of L-dopa medication. The proposed method provides a robust, accurate, and practical method for the assessment of brain connectivity patterns from functional neuroimaging data.

## 1. Introduction

The interaction between macroscopic brain regions has been increasingly recognized as being vital for understanding the normal brain function and the pathophysiology of many neuropsychiatric diseases. Brain connectivity patterns derived from neuroimaging methods are therefore of great interest, and several recently published reviews have described different modeling methods for inferring brain connectivity from fMRI data [1, 2]. Specifically, graphical models which represent statistical dependence relationships between time series derived from brain regions, such as structural equation models [3], dynamic causal models [4], and Bayesian networks [5], appear to be well suited for assessing connectivity between brain regions.

Graphical models, when applied to functional neuroimaging data, represent brain regions of interest (ROIs) as nodes and the stochastic interactions between ROIs as edges. However, in most nonbrain imaging graphical model applications, the primary goal is to create a model that fits the overall multivariate data well, does not necessarily accurately reflect the particular connections between nodes. Yet in the applications of graphical models to brain connectivity, the neuroscientific interpretation is largely based on the pattern of connections inferred by the model. This places a premium on accurately determining the “inner workings” of the model such as accounting for the error rate of the edges in the model.

The false discovery rate (FDR) [6, 7], defined as the expected ratio of spurious connections to all learned connections, has been suggested as a suitable error-rate control criterion when inferring brain connectivity. Compared with traditional type I and type II statistical error rates, the FDR is more informative in bioinformatics and neuroimaging, since it is directly related with the uncertainty of the reported positive results. When selecting candidate genes for genetic research, for example, researchers may want 70% of selected genes to be truly associated with the disease, that is, an FDR of 30%.

Naively controlling traditional type I and type II error rates at specified levels may not necessarily result in reasonable FDR rates, especially in the case of large, sparse networks. For example, consider an undirected network with 40 nodes, with each node interacting, on average, with 3 other nodes; that is, there are 60 edges in the network. An algorithm with the *realized* type I error rate of 5% and the *realized* power of 90% (i.e., the *realized* type II error rate = 10%) will recover a network with 60 × 90% = 54 correct connections and [40 × (40 − 1)/2 − 60] × 5% = 36 false connections, which means that 36/(36 + 54) = 40% of the claimed connections actually would not exist in the true network! This example, while relatively trivial, demonstrates that the FDR may not be kept suitably low by simply controlling traditional type I and type II error rates.

Recent work in the machine learning field has started to investigate controlling the FDR in network structures using a generic Bayesian approach and classical FDR assessment [8]. This work was subsequently extended to look specifically at graphical models where the FDR was assessed locally at each node [9].

Li and Wang proposed a network-learning method that allows asymptotically control of the FDR globally. They based their approach on the PC algorithm (named after Peter Spirtes and Clark Glymour), a computationally efficient and asymptotically reliable Bayesian network-learning algorithm. The PC algorithm assesses the (non)existence of an edge in a graph by determining the conditional dependence/independence relationships between nodes [10]. However, different from the original PC algorithm, which controls the type I error rate individually for each edge during conditional independence testing, the Li and Wang algorithm, referred as the PC_fdr_ algorithm, is capable of asymptotically controlling the FDR under prespecified levels [11]. The PC_fdr_ algorithm does this by interpreting the learning of a network as testing the existence of edges, and thus the FDR control of edges becomes a multiple-testing problem, which has a strong theoretical basis and has been extensively studied by statisticians [11].

Beside giving an introduction of these recent advancements, this paper will present two extensions to the original PC_fdr_ algorithm, the combination of which leads to a multisubject brain connectivity modeling approach incorporating FDR control, *a priori* knowledge and group inference. One extension is an adaptation of *a priori* knowledge, allowing users to specify which edges must appear in the network, which cannot and which are to be learned from data. The resulting algorithm is referred to as PC_fdr_^+^ algorithm in this paper. Many applications require imposing prior knowledge into network learning. For example, analyzing causal relationship in time series may forbid backward connections from time *t* + 1 to *t*, such as that in dynamic Bayesian networks. In some situations, researchers may want to exclude some impossible connections based on anatomical knowledge. Incorporating *a priori* knowledge into PC_fdr_ algorithm allows for more flexibility in using the method and potentially leads to greater sensitivity in accurately discovering the true brain connectivity.

The second extension to PC_fdr_ algorithm is a combination of the PC_fdr_ algorithm and a mixed-effect model to robustly deal with intersubject variability. As neuroimaging research typically involves a group of subjects rather than focusing on an individual subject, group analysis plays an important role in final biological interpretations. However, compared with the extensive group-level methods available for analysis of amplitude changes in blood-oxygen-level-dependent (BOLD) signals (e.g., Worsley et al. [12], Friston et al. [13]), the problem of group-level brain connectivity analysis is less well studied. This is likely due to the fact that it requires not only accommodating the variances and the correlations across subjects, but also accounting for the potentially different structures of subject-specific brain connectivity networks. The proposed group-level exploratory approach for brain connectivity inference combines the PC_fdr_ algorithm (or the extended PC_fdr_^+^ algorithm if *a priori* knowledge is available) and a mixed-effect model, a widely used method for handling intersubject variability.

Several methods have been proposed to infer group connectivity in neuroimaging. Bayesian model selection [14] handles intersubject variability and error control; however, its current proposed implementation does not scale well, making it more suitable for confirmatory, rather than an exploratory research. Varoquaux et al. [15] propose a data-driven method to estimate large-scale brain connectivity using Gaussian modeling and deals with the variability between subjects by using optimal regularization schemes. Ramsey et al. [16] describe and evaluate a combination of a multisubject search algorithm and the orientation algorithm.

The major distinguishing feature of the proposed approach compared to these aforementioned approaches is that the current data-driven approach aims at controlling the FDR directly at the group-level network. We demonstrate that in simulations that, with a sufficiently large subject size, the proposed group-level algorithm is able to reliably recover network structures and still control the FDR around prespecified levels. When the proposed approach, referred as the gPC_fdr_^+^ algorithm, is applied to real fMRI data with Parkinson's disease, we demonstrate evidence of direct and indirect (i.e., compensatory) disease-related connectivity changes, as well as evidence that L-dopa provides a “normalizing” effect on connectivity in Parkinson's disease, consistent with its dramatic clinical effect.

## 2. Materials and Methods

### 2.1. Preliminaries

Graphical models, such as Bayesian networks, encode conditional independence/dependence relationships among variables graphically with nodes and edges according to the Markov properties [17]. The concept of conditional (in)dependence is very important for the inference of brain connectivity, as it assists in distinguishing between direct and indirect connectivity. For example, the activities in two brain regions are initially correlated, but become independent after all possible influences from other brain regions are removed, then this is an example of indirect connectivity, as the initial activity was actually induced by common input from another region(s). On the other hand, if the activities of two brain regions are correlated even after all possible influences from other regions are removed, then very likely there is a direct functional connection between them and hence is an example of direct connectivity. Conditional dependence is the real interest in learning brain connectivity because it implies that two brain regions are directly connected.

Since a graphical model is a graphical representation of conditional independence/dependence relationships, the nonadjacency between two nodes is tested by inspecting their conditional independence given all other nodes. As multiple edges are tested simultaneously, FDR-control procedures should be applied to correct the effect of multiple testing.

Given two among *N* random variables, there are 2^*N*−2^ possible subsets of the other *N* − 2 variables upon which the two variables could be conditionally independent. To avoid exhaustively testing such an exponential number of conditional independence relationships, the following proposition [10] can be employed [9, 11].

**Proposition 1**

Given a multivariate probability distribution whose conditional independence relationships can be perfectly encoded as a Bayesian network according to the Markov property, two nodes *a* and *b* are nonadjacent if and only if there is a subset *C* of nodes either all in the neighbors of *a* or all in the neighbors of *b* such that *a* and *b* are conditionally independent on given *C*.

Based on Proposition 1, nodes *a* and *b* can be disconnected once they are found conditionally independent upon a conditional node set *C*. As the tests of adjacency progress for every node pair, the neighborhood of nodes keeps shrinking, so an exhaustive search of the conditional node set *C* is avoided. This greatly reduces computation, especially for a sparse network.

### 2.2. Brain Connectivity Inference Incorporating False Discovery Rate Control and *A Priori* Knowledge

#### 2.2.1. PC_fdr_^+^ Algorithm

The initial version of Li and Wang's [11] method, called the PC_fdr_ algorithm, was proved to be capable of asymptotically controlling the FDR. Here we present an extension of the PC_fdr_ algorithm which can incorporate *a priori* knowledge, which was not specified in the original PC_fdr_ algorithm. We name the extension as the PC_fdr_^+^ algorithm where the superscript “+” indicates that it is an extension. The pseudocode of the PC_fdr_^+^ algorithm is given in Algorithm 1, and its Matlab implementation is downloadable at http://www.junningli.org/software. It handles prior knowledge with two inputs: *E*_must_, the set of edges assumed to appear in the true graph, and *E*_test_, the set of edges to be tested from the data. The original PC_fdr_ algorithm can thus be regarded as a special case of the extended algorithm, by setting *E*_must_ = *∅* and *E*_test_ = {all  possible  edges}.

#### 2.2.2. Asymptotic Performance

Before we present theorems about the asymptotic performance of the PC_fdr_^+^ algorithm and its heuristic modification, let us first introduce the assumptions related to the theorems.

(A1) The multivariate probability distribution *P* is faithful to a directed acyclic graph (DAG) whose skeleton is *G*_true_.
(A2) The number of vertices is fixed.
(A3) Given a fixed significance level of testing conditional independence, the power of detecting conditional dependence approaches 1 at the limit of large sample sizes.
(A4) The union of *E*_must_, the edges assumed to be true, and *E*_test_, the edges to be tested, covers *E*_true_, all the true edges; that is, *E*_test_ ∪ *E*_must_⊇*E*_true_.

Assumption (A1) is generally assumed when graphical models are applied, and it restricts the probability distribution *P* to a certain class. Assumption (A2) is usually implicitly stated, but here we emphasize it because it simplifies the proof. Assumption (A3) may seem overly restrictive, but actually can be easily satisfied by standard statistical tests, such as the likelihood ratio test introduced by Neyman and Pearson [18] and the partial-correlation test by Fisher [19], if the data are identically and independently sampled. Assumption (A4) relates to prior knowledge, which interestingly does not require that the assumed “true” edges *E*_must_ be a subset of the true edges *E*_true_, but just that all true edges are included in the union of the assumed “true” edges and the edges to be tested.

The detection power of the PC_fdr_^+^ algorithm and its heuristic modification at the limit of large sample sizes is elucidated in Theorem 2.

**Theorem 2**

Assuming (A1), (A2), and (A3), both the *PC*_*fdr*_^+^ algorithm and its heuristic modification, the *PC*_fdr*_^+^ algorithm, are able to recover all the true connections in *E*_*test*_ with probability one as the sample size approaches infinity:

(1) $$\begin{array}{c} \underset{m\to \infty }{\lim}P\left(E_{true}^{\prime }\subseteq E_{stop}^{\prime }\right)=1, \end{array}$$

where *E*_*true*_′ denotes the set of true edges in *E*_*test*_; that is, *E*_*true*_′ = *E*_*true*_∩*E*_*test*_, *E*_*stop*_′ denotes the set of edges inferred by the algorithm about *E*_*test*_; that is, *E*_*stop*_′ = *E*_*stop*_∩*E*_*test*_, and *m* denotes the sample size.

It should be noted that Theorem 2 does not need Assumption (A4), which implies that the true edges in *E*_test_ are still able to be recovered by the algorithms with probability one at the limit of large sample sizes, even if the edges assumed to be present by users are not completely correctly specified.

The FDR of the PC_fdr_^+^ algorithm at the limit of large sample sizes is elucidated in Theorem 3.

**Theorem 3**

Assuming (A1), (A2), (A3), and (A4), the FDR of the set of edges inferred by the *PC*_*fdr*_^+^ algorithm about *E*_*test*_ approaches a value not larger than the user-specified level *q* as the sample size *m* approaches infinity:

(2) $$\begin{array}{c} \underset{m\to \infty }{\mathrm{limsup}}\;FDR\left(E_{stop}^{\prime },E_{true}^{\prime }\right)\leq q, \end{array}$$

where *FDR*(*E*_*stop*_′, *E*_*true*_′) is defined as

(3) $$\begin{array}{c} FDR\left(E_{stop}^{\prime },E_{true}^{\prime }\right)=E\left[\frac{\left|E_{stop}^{\prime }\setminus E_{true}^{\prime }\right|}{\left|E_{stop}^{\prime }\right|}\right],\\ Define\;\;\frac{\left|E_{stop}^{\prime }\setminus E_{true}^{\prime }\right|}{\left|E_{stop}^{\prime }\right|}=0,\;if\;\;E_{stop}^{\prime }=\emptyset . \end{array}$$

Theorem 3 concerns the PC_fdr_^+^ algorithm, and it requires Assumption (A4). We are still not sure whether similar FDR performance can be proved for the PC_fdr*_^+^ algorithm. Assumption (A4) does not require that the assumed “true” edges *E*_must_ is a subset of the true edges *E*_true_ but only that all true edges are included in the union of the assumed “true” edges and the edges to be tested. This is particularly useful in practice, since it does not require users' prior knowledge to be absolutely correct, but allows some spurious edges to be involved in *E*_must_, once all true edges have been included in either *E*_must_ or *E*_test_. Assumption (A4) can be satisfied by making *E*_test_ ∪ *E*_must_ large enough to cover all the true edges, but as shown in (4) this will increase the computational cost of the algorithm.

Theorems 2 and 3 address the performance of the PC_fdr_^+^ algorithm and its heuristic modification at the limit of large sample sizes. Because the PC_fdr_^+^ algorithm is derived from the PC_fdr_ algorithm, its performance should be very similar. The numerical examples of the PC_fdr_ algorithm in Li and Wang's [11] work may provide helpful and intuitive understanding on the performance of the PC_fdr_^+^ algorithm with moderate sample sizes.

The detailed proofs of Theorems 2 and 3 are provided in Appendix A.

#### 2.2.3. Computational Complexity

The majority of the computational effort in the PC_fdr_^+^ is utilized in performing statistical tests of conditional independence at step 7 and the FDR at step 11. If the algorithm stops at the depth *d* = *d*_max_, then the number of conditional independence tests required is bounded by

(4) T=2|Etest|∑d=0dmaxCΔ−1d≤|Etest|2Δ,

where |*E*_test_| is the number of edges to be tested, Δ is the maximum degree of graph *G*_init_ (the graph formed at step 1) whose edges are *E*_must_∩*E*_test_, and *C*_Δ−1_^*d*^ is the number of combinations of choosing *d* unordered and distinct elements from Δ − 1 elements. The bound usually is very loose, because it assumes that no edge has been removed until *d* = *d*_max_.

The computational complexity of the FDR procedure, Algorithm 2, invoked at step 11 of the PC_fdr_^+^ algorithm is *O*(*H*log(*H*)) when it is invoked for the first time, where *H* = |*E*_test_| is the number of input *p* values and is *O*(*H*) later, with the optimization suggested in Appendix B. In the worst case that *p*_*a*⊥*b*∣*C*_ is always larger than *p*_*a*~*b*_^max^, the complexity of the computation spent on the FDR control in total is bounded by *O*(|*E*_test_|log(|*E*_test_|) + *T*|*E*_test_|) where *T* is the number of performed conditional independence tests (see (4)). This is a very loose bound because it is rare that *p*_*a*⊥*b*∣*C*_ is always larger than *p*_*a*~*b*_^max^.

In practice, the PC_fdr_^+^ algorithm runs very quickly, especially for sparse networks. In our experiments (see Section 3.1), it took about 10 seconds to infer the structure of a first-order dynamic network with 20 nodes from data of 1000 time points.

#### 2.2.4. Miscellaneous Discussions

It should be noted that controlling the FDR locally is not equivalent to controlling it globally. For example, if it is known that there is only one connection to test for each node, then controlling the FDR locally in this case will degenerate to controlling the point-wise error rate, which cannot control the FDR globally.

Listgarten and Heckerman [8] proposed a permutation method to estimate the number of spurious connections in a graph learned from data. The basic idea is to repetitively apply a structure learning algorithm to data simulated from the null hypotheses with permutation. This method is generally applicable to any structure learning method, but permutation may make the already time-consuming structure learning problem even more computationally cumbersome, limiting its use in practical situations.

### 2.3. FDR-Controlled Group Brain Connectivity Inference with or without *A Priori* Knowledge

In this section, we propose another extension to the PC_fdr_ algorithm: from the single subject level to the group level. Assessing group-level activity is done by considering a mixed-effect model (Step 7 of Algorithm 3), and we name it the gPC_fdr_ algorithm where “g” indicates that it is an extension at the group level. When also incorporating *a priori* knowledge, the resulting algorithm is named the gPC_fdr_^+^ algorithm.

Suppose we have *m* subjects within a group. Then for subject *i*, the conditional independence between the activities of two brain regions *a* and *b* given other regions *C* can be measured by the partial correlation coefficient between *X*_*a*_(*i*) and *X*_*b*_(*i*) given *X*_*C*_(*i*), denoted as *r*_*ab*∣*C*_(*i*). Here *X*_•_ denotes variables associated with a vertex or a vertex set, and index *i* indicates that these variables are for subject *i*. By definition, the partial correlation coefficient *r*_*ab*∣*C*_(*i*) is the correlation coefficient between the residuals of projecting *X*_*a*_(*i*) and *X*_*b*_(*i*) onto *X*_*C*_(*i*) and can be estimated by the sample correlation coefficient as

(5) r^ab ∣ C(i)=Cov[Ya ∣ C(i),Yb ∣ C(i)]Var[Ya ∣ C(i)]Var[Yb ∣ C(i)],

where

(6) $$\begin{array}{c} \beta_{a\;|\;C}\left(i\right)=\arg\underset{\beta }{\min}{\left|X_{a}\left(i\right)-X_{C}\left(i\right)\beta \right|}^{2},\\ \beta_{b\;|\;C}\left(i\right)=\arg\underset{\beta }{\min}{\left|X_{b}\left(i\right)-X_{C}\left(i\right)\beta \right|}^{2},\\ Y_{a\;|\;C}\left(i\right)=X_{a}\left(i\right)-X_{C}\left(i\right)\beta_{a\;|\;C}\left(i\right),\\ Y_{b\;|\;C}\left(i\right)=X_{b}\left(i\right)-X_{C}\left(i\right)\beta_{b\;|\;C}\left(i\right). \end{array}$$

For clarity, in the following discussion we omit the subscript “*ab* | *C*” and simply use index “*i*” to emphasize that a variable is associated with subject *i*.

To study the group-level conditional independence relationships, a group-level model should be introduced for *r*_*i*_. Since partial correlation coefficients are bounded and their sample distributions are not Gaussian, we apply Fisher's *z*-transformation to convert (estimated) partial correlation coefficients *r* to a Gaussian-like distributed *z*-statistic *z*, which is defined as

(7) $$\begin{array}{c} z=Z\left(r\right)=\frac{1}{2}\ln\left(\frac{1+r}{1-r}\right), \end{array}$$

where *r* is a (estimated) partial correlation coefficient and *z* is its *z*-statistic.

The group model we employ is

(8) $$\begin{array}{c} z_{i}=z_{g}+e_{i}, \end{array}$$

where *e*_*i*_ follows a Gaussian distribution *N*(0, *σ*_*g*_^2^) with zero mean and *σ*_*g*_^2^ variance. Consequently, the group-level testing of conditional independence is to be used to test the null hypothesis *z*_*g*_ = 0.

Because *z*_*i*_ is unknown and can only be estimated, the inference of *z*_*g*_ should be conducted with ${\hat{z}}_{i}=Z({\hat{r}}_{i})$. If *X*_*a*_(*i*), *X*_*b*_(*i*), and *X*_*C*_(*i*) jointly follow a multivariate Gaussian distribution, then ${\hat{z}}_{i}$ asymptotically follows a Gaussian distribution *N*(*z*_*i*_, *σ*_*i*_^2^) with *σ*_*i*_^2^ = 1/(*N*_*i*_ − *p* − 3), where *N*_*i*_ is the sample size of subject *i*'s data and *p* represents the number of variables in *X*_*C*_(*i*). Therefore, based on (8), we have

(9) $$\begin{array}{c} {\hat{z}}_{i}=z_{g}+e_{i}+\epsilon_{i}, \end{array}$$

where *ϵ*_*i*_ follows *N*(0, *σ*_*i*_^2^) and *e*_*i*_ follows *N*(0, *σ*_*g*_^2^). This is a mixed-effect model where *ϵ*_*i*_ denotes the intrasubject randomness and *e*_*i*_ denotes the intersubject variability. At the group level, ${\hat{z}}_{i}$ follows a Gaussian distribution *N*(*z*_*g*_, *σ*_*i*_^2^ + *σ*_*g*_^2^). Note that unlike regular mixed-effect models, the intrasubject variance *σ*_*i*_^2^ in this model is known, because *N*_*i*_ and *p* are known given the data *X*(*i*) and *C*. In general, *σ*_*i*_^2^ = 1/(*N*_*i*_ − *p* − 3) is not necessarily equal to *σ*_*j*_^2^ for *i* ≠ *j*, and the inference of *z*_*g*_ should be conducted in the manner of mixed models, such as estimating *σ*_*g*_^2^ with the restricted maximum likelihood (ReML) approach. However, if the sample size of each subject's data is the same, then *σ*_*i*_^2^ equals *σ*_*j*_^2^. For this balanced case, which is typically true in fMRI applications and as well the case in this paper, we can simply apply a *t*-test to ${\hat{z}}_{i}$'s to test the null hypothesis *z*_*g*_ = 0.

Replacing Step 7 of the single-subject PC_fdr_ algorithm (i.e., the intrasubject hypothesis test) with the test of *z*_*g*_ = 0, we can extend the single-subject version of the algorithm to its group-level version. We will employ this *t*-test in our simulations and in the real fMRI data analysis presented later in this paper. Such a testing approach significantly simplifies the estimation process, and our simulation results presented later demonstrate that this method can still control the FDR at a user specified error rate level.

## 3. Experiments

### 3.1. Simulations for the PC_fdr_^+^ Algorithm

**Figure 1:**
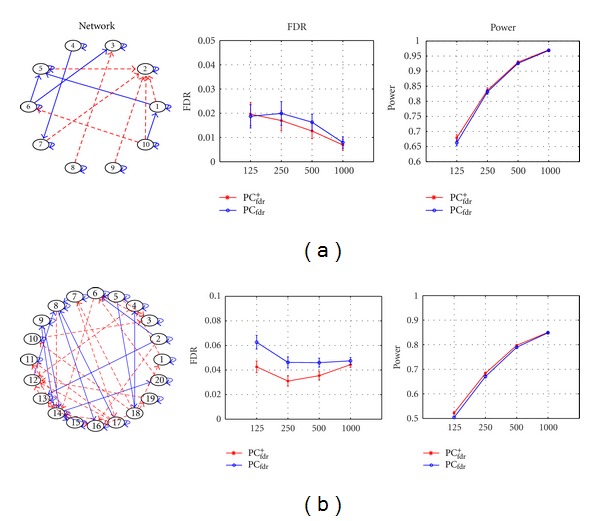
Simulation results for the PC_fdr_^+^ algorithm. (a) Simulation results for the network with 10 nodes and 23 edges. (b) Simulation results for the network with 20 nodes and 56 edges. In the networks, solid arrows represent edges from time *t* to *t* + 1, and dashed arrows represent edges with no time lag (i.e., from time *t* to *t*). For the FDR and detection power curves, the blue solid lines represent the PC_fdr_ algorithm, the red solid lines represent the PC_fdr_^+^ algorithm, the *x*-axis means the sample sizes, and the *y*-axis means the FDR or detection power.

 Here we compare the performances of the proposed PC_fdr_^+^ algorithm and the original PC_fdr_ algorithm, using time series generated from two dynamic Bayesian networks in Figure 1. One network has 20 nodes (10 channels) and 23 edges, and the other has 40 nodes (20 channels) and 56 edges. The dynamic Bayesian networks are assumed Gaussian, with connection coefficients uniformly distributed in [0.2,0.6] with Gaussian noise whose amplitudes are uniformly distributed in [0.5, 1.1]. We use partial correlation coefficients to test conditional independence relationships. The target FDR for both methods is set as 5%. For the PC_fdr_^+^ algorithm, one-third of the nonexisting connections are excluded as prior knowledge.

Figure 1 shows the estimated FDR and detection power results, at sample sizes of 125, 250, 500, and 1000 time points and with 50 repetitive trials for each sample size. As shown in graphs (a) and (b), the PC_fdr_^+^ and PC_fdr_ algorithms can both control the FDR under or around 5%. For both methods, the detection power increases as the sample size increases. However, we can see that the PC_fdr_^+^ algorithm yields higher detection power and lower FDR than the original PC_fdr_ algorithm does. As mentioned earlier in the Introduction Section, the PC_fdr_^+^ algorithm has the advantage of providing researchers more flexibility in using the method and higher accuracy in discovering brain connectivity.

### 3.2. Simulations for the gPC_fdr_ Algorithm

The simulations here serve two purposes: first, to verify whether the proposed gPC_fdr_ algorithm for modeling brain connectivity can control the FDR at the group level, and second, to compare the gPC_fdr_ algorithm with the single-subject PC_fdr_ algorithm proposed in [11] and the state-of-art IMaGES algorithm investigated in Ramsey et al. [16] for inferring the structure of the group connectivity network.

The simulations were conducted as follows. First, a connectivity network is generated as the group-level model. Individual subject-level networks are then derived from the group-level model by randomly adding or deleting connections with a small probability, and subject-specific data are generated according to individual subject networks. Next, the network-learning methods, that is, the proposed gPC_fdr_ algorithm, the single-subject PC_fdr_ method with pooling together the data from all subjects, and the IMaGES algorithm, are applied to the simulated data. Finally, the outputs of the algorithms are compared with the true group-level network to evaluate their accuracy.

The data generation process is as follows.

1. Randomly generate a directed acyclic graph (DAG) as the group-level network and associate each connection with a coefficient. The DAG is generated by randomly connecting nodes with edges and then orienting the edges according to a random order of the nodes. The connection coefficients are assigned as random samples from the uniform distribution *U*(*β*_1_, *β*_2_), where *β*_1_ and *β*_2_ characterize the coefficient strength.
2. For each subject, a subject-level network is derived from the group-level network by randomly adding and deleting connections. More specifically, for each of the existing connections, the connection is deleted with probability 0.05, and for each of the absent connections, a connection is added with probability 0.01. The corresponding connection coefficients are randomly sampled from the uniform distribution *U*(*β*_1_, *β*_2_).
3. Given a subject-level network, the subject-specific data are generated from a Gaussian Bayesian network, with the additional Gaussian noise following the standard Gaussian distribution *N*(0,1).

**Figure 2:**
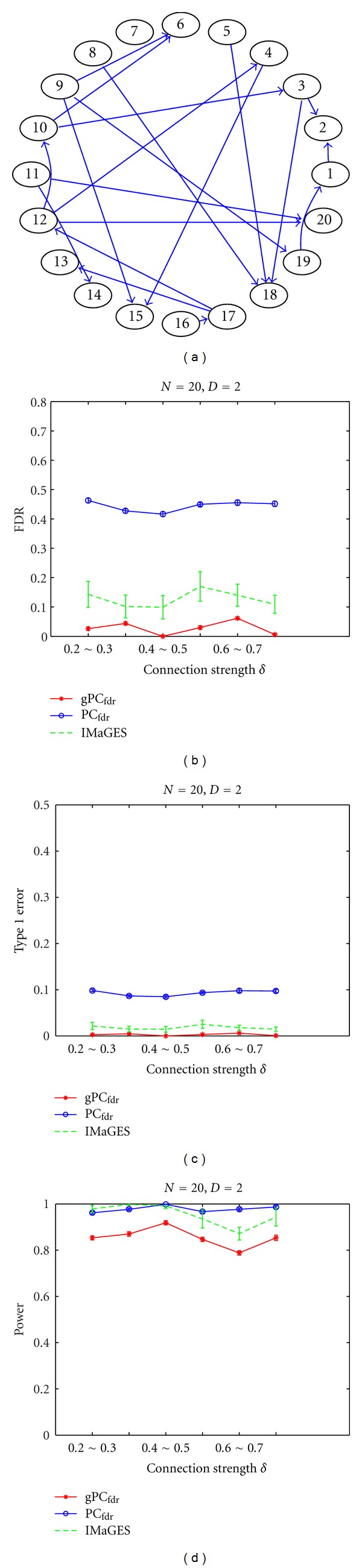
Simulation 1: assessing the effects of connection strength on the learned group networks. (a) The group-level network, with 20 nodes and an average of two connections per node. (b) The FDR curves (with standard deviation marked) of the gPC_fdr_ algorithm, the original PC_fdr_ algorithm by pooling all subject data together, and the IMaGES algorithm. (c) The type I error rate curves. (d) The detection power curves. The *x*-axis represents the generating distribution *U*(*β*_1_, *β*_2_) for sampling the connection coefficients.

In the first simulation, we compare the performances of the proposed gPC_fdr_ algorithm, the original PC_fdr_ algorithm, and the IMaGES algorithm [16], when using different connection coefficient strengths. In this example, the group-level network is the DAG in Figure 2(a). From this model, twenty subject-level models are derived, and for each subject, data with three hundred samples are simulated. To test the performances of the algorithms with a range of connection strengths, we vary the connection coefficient generating distribution *U*(*β*_1_, *β*_2_) gradually from *U*(0.2, 0.3) to *U*(0.7, 0.8). At the network-learning stage, we set the target FDR to be 5% for the gPC_fdr_ algorithm. For reliable assessment, this procedure is repeated thirty times.

Figures 2(b), 2(c), and 2(d) show the FDR and the type I error rate, and the detection power results as a function of connection strength. We note that all methods are relatively invariant to connection strength. The proposed gPC_fdr_ algorithm steadily controls the FDR below or around the desired level and accurately makes the inference at the group level. The detection power of IMaGES algorithm is higher than that of gPC_fdr_ algorithm, but it fails to control the FDR under the specified 5% level. Its higher detection power is achieved by sacrificing FDR. This is reasonable, since IMaGES is not specifically designed to control the FDR error rate.

**Figure 3:**
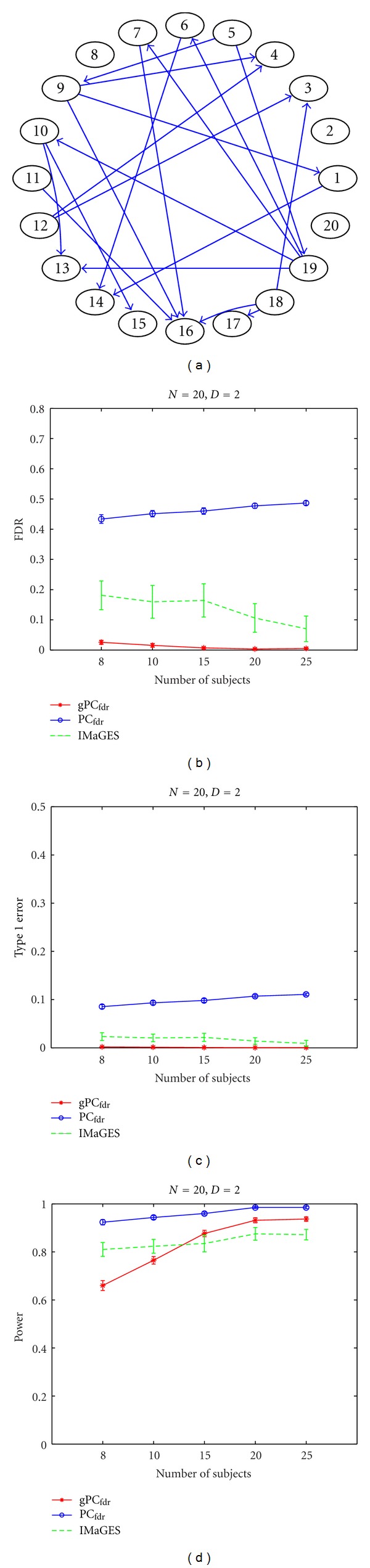
Simulation 2: assessing the effects of increasing the number of subjects on the learned group networks. (a) The group-level network, with 20 nodes and an average of two connections per node. (b) The FDR curves (with standard deviation marked) of the proposed gPC_fdr_ algorithm, the original PC_fdr_ algorithm by pooling all subject data together, and the IMaGES algorithm. (c) The type I error rate curves. (d) The detection power curves. The *x*-axis represents the number of subjects within the group.

In the second simulation, we test the performances of the algorithms as a function of the number of subjects within the group. The group-level network is the DAG in Figure 3(a), and the number of subjects increases from eight to twenty-five. At the network-learning stage, we set the target FDR to be 5%. This procedure is repeated thirty times.

Figure 3(b) demonstrates the FDR results as a function of the number of subjects within the group. It is noted that the proposed gPC_fdr_ algorithm is able to keep the FDR below or around the specified level. The detection power gradually increases as the number of subjects increases. When there are more than 15 subjects, the gPC_fdr_ algorithm seems that it can achieve higher (better) detection power and lower (better) FDR and type I error rate than the IMaGES algorithm does. It suggests that when the number of subjects is large enough, the proposed gPC_fdr_ algorithm can jointly address efficiency, accuracy, and intersubject variability. The original PC_fdr_ algorithm of simply pooling the data together fails to control the FDR, and the resulting FDR does not decrease as the number of subject increases, probably due to the increasing heterogeneity within the group. In order to investigate the effects of the number of ROIs, we also investigate two networks with 15 and 25 nodes, respectively, and repeat the simulations (not shown here). The results are qualitatively similar to what we show here.

### 3.3. fMRI Application

In order to assess the real-world application performance of the proposed method, we apply the gPC_fdr_^+^ algorithm for inferring group brain connectivity network to fMRI data collected from twenty subjects. All experiments were approved by the University of British Columbia Ethics Committee. Ten normal people and ten Parkinson's disease (PD) patients participated in the study. During the fMRI experiment, each subject was instructed to squeeze a bulb in their right hand to control an “inflatable” ring so that it smoothly passed through a vertically scrolling a tunnel. The normal controls performed only one trial, while Parkinson's subjects performed twice, once before L-dopa medication and the other approximately an hour later, after taking medication.

Three groups were categorized: group N for the normal controls, group P_pre_ for the PD patients before medication, and group P_post_ for the PD patients after taking L-dopa medication. For each subject, 100 observations were used in the network modeling. For details of the data acquisition and preprocessing, please refer to Palmer et al. [20]. 12 anatomically defined regions of interest (ROIs) were chosen based on prior knowledge of the brain regions associated with motor performance (Table 1).

**Figure 4:**
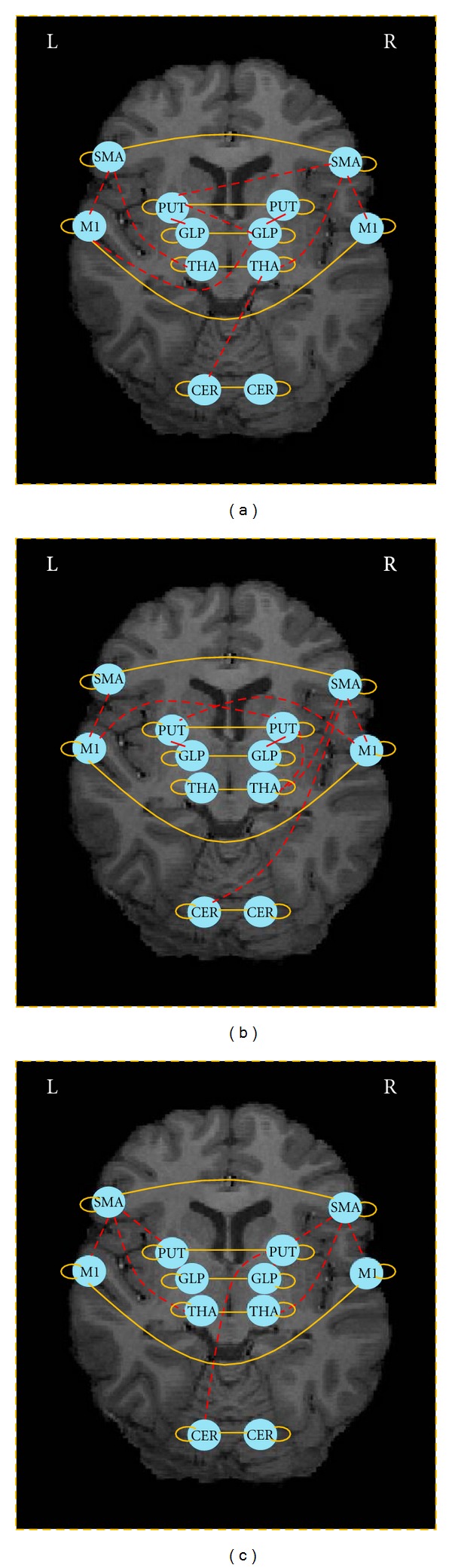
(a) Learned brain connectivity for the normal group (group N). (b) Learned brain connectivity for the PD group before medication (group P_pre_). (c) Learned brain connectivity for the PD group after medication (group P_post_). Here “L” and “R” refer to the left and right sides, respectively. The solid lines are predefined connectivity, and the dashed lines are learned connectivity.

We utilized the two extensions of the PC_fdr_ algorithm and learned the structures of first-order group dynamic Bayesian networks from fMRI data. Because the fMRI BOLD signal can be considered as the convolution of underlying neural activity with a hemodynamic response function, we assumed that there must be a connection from each region at time *t* to its mirror at time *t* + 1. We also assumed that there must be a connection between each region and its homologous region in the contralateral hemisphere. The TR interval (i.e., sampling period) was a relatively long, 1.985 seconds; we restricted ourselves to learn only connections between ROIs without time lags. In total, there are 12 + 6 = 18 pre-defined connections and 12 × (12 − 1) ÷ 2 − 6 = 60 candidate connections to be tested. The brain connectivity networks (with the target FDR of 5%) learned for the normal (group N) and PD groups before (group P_pre_) and after (group P_post_) medication are compared in Figure 4. Note the connection between the cerebellar hemisphere and contralateral thalamus in the normal subjects and between the supplementary motor area (SMA) and the contralateral putamen, consistent with prior knowledge. Interestingly, in P_pre_ subjects, the left cerebellum now connects with the right SMA, and the right SMA ↔ left putamen connection is lost. Also, there are now bilateral primary motor cortex (M1) ↔ putamen connections seen in the P_pre_ group, presumably as a compensatory mechanism. After medication (P_post_), the left SMA ↔ left thalamus connection is restored back to be normal.

## 4. Discussion

Up to now, graphical models to infer brain connectivity from fMRI data have implicitly relied on the unrealistic assumption that if a model accurately represented the overall activity in several ROIs, the internal connections of such a model would accurately reflect underlying brain connectivity. The PC_fdr_ algorithm was designed to loosen this overly restrictive assumption and asymptotically control the FDR of network connections inferred from data.

In this paper, we first presented the PC_fdr_^+^ algorithm, an extension of the PC_fdr_ algorithm, which allows for incorporation of prior knowledge of network structure into the learning process, greatly enhancing its flexibility in practice. The PC_fdr_^+^ algorithm handles prior knowledge with two inputs: *E*_must_, which is the set of edges that are assumed to appear in the true graph, and *E*_test_, the set of edges that are to be tested from the observed data. We proved that, with mild assumptions and at the limit of large samples, the PC_fdr_^+^ algorithm is able to recover all the true edges in *E*_test_ and also curb the FDR of the edges inferred about *E*_test_.

It is interesting that the PC_fdr_^+^ algorithm does not require the assumed “true” edges *E*_must_ to be a subset of the true edges *E*_true_, but only that all true edges are included in the union of the assumed “true” edges and the edges to test. This is very useful in research practice, since it allows some spurious edges to be involved in *E*_must_, as long as all the true edges have been included in either *E*_must_ or *E*_test_. Users can satisfy this requirement by making *E*_test_ ∪ *E*_must_ large enough to cover all the true edges.

When we compared the PC_fdr_^+^ algorithm with the original PC_fdr_ algorithm, both of them successfully controlled the FDR under the target threshold in simulations, providing a practical tradeoff between computational complexity and accuracy. However, the PC_fdr_^+^ algorithm achieved better detection power and better FDR than the original PC_fdr_ algorithm. Incorporating prior knowledge into PC_fdr_ algorithm therefore enhances inference accuracy and improves the flexibility in using the method.

Another extension to PC_fdr_ algorithm we described here was the ability to infer brain connectivity patterns at the group level, with intersubject variance explicitly taken into consideration. As a combination of the PC_fdr_ algorithm and a mixed-effect model, the gPC_fdr_ algorithm takes advantage of the error control ability of the PC_fdr_ algorithm and the capability of handling intersubject variance. The simulation results suggest that the proposed method was able to accurately discover the underlying group network and steadily control the false discovery rate. Moreover, the gPC_fdr_ algorithm was shown to be much more reliable than simply pooling together the data from all subjects. This may be especially important in disease states and older subjects. Compared with the IMaGES algorithm, gPC_fdr_ demonstrated better control of the FDR.

As with all group models, a limitation of the proposed gPC_fdr_ algorithm is the requirement of a sufficient number of subjects. While it is appreciated that in many biomedical applications data collection is resource intensive, and if the number of subjects is insufficient, the gPC_fdr_ algorithm may give unreliable results. Nevertheless, the group extension to the PC_fdr_ algorithm is one attempt to make brain connectivity inference using error-rate-controlled exploratory modeling.

When applying the proposed gPC_fdr_^+^ to fMRI data collected from PD subjects performing a motor tracking task, we found group evidence of disease changes (e.g., loss of left cerebellar ↔ SMA connectivity), compensatory changes in PD (e.g., bilateral M1 ↔ contralateral putamen connectivity), and evidence of restoration of connectivity after medication (left SMA ↔ left thalamus). The tremendous variability in clinical progression of PD is likely due to variability not only in disease rate progression, but also in variability in the magnitude of compensatory changes. This highlights the importance of the proposed method, as it allows robust estimation of disease effects, compensatory effects, and effects of medication, all with a reasonable sample size, despite the enhanced intersubject variability seen in PD.

## Figures and Tables

**Algorithm 1:**
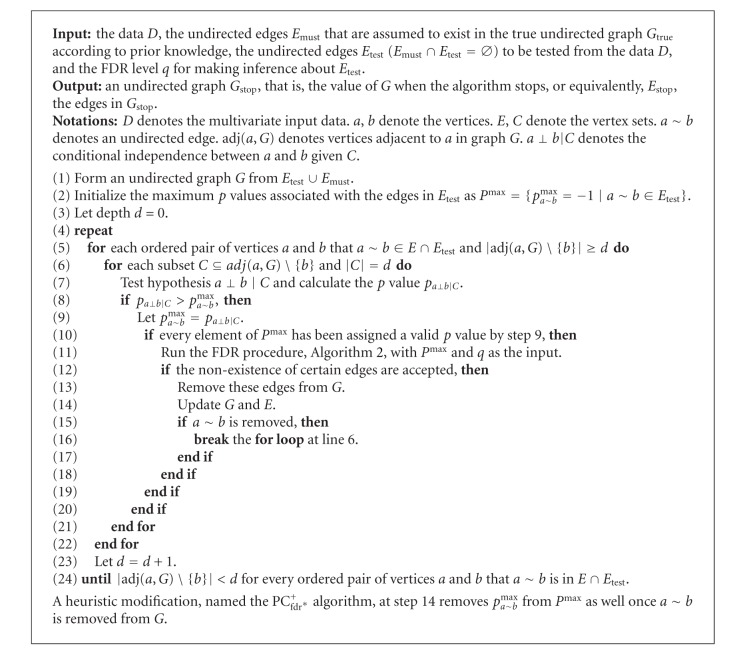
The PC_fdr_^+^ algorithm.

**Algorithm 2:**
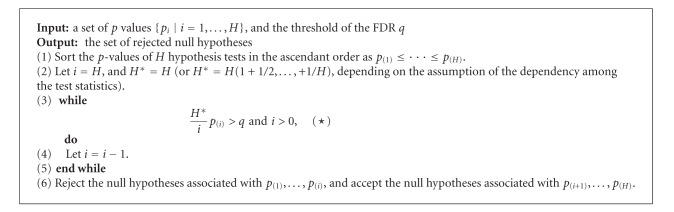
FDR setup [6].

**Algorithm 3:**
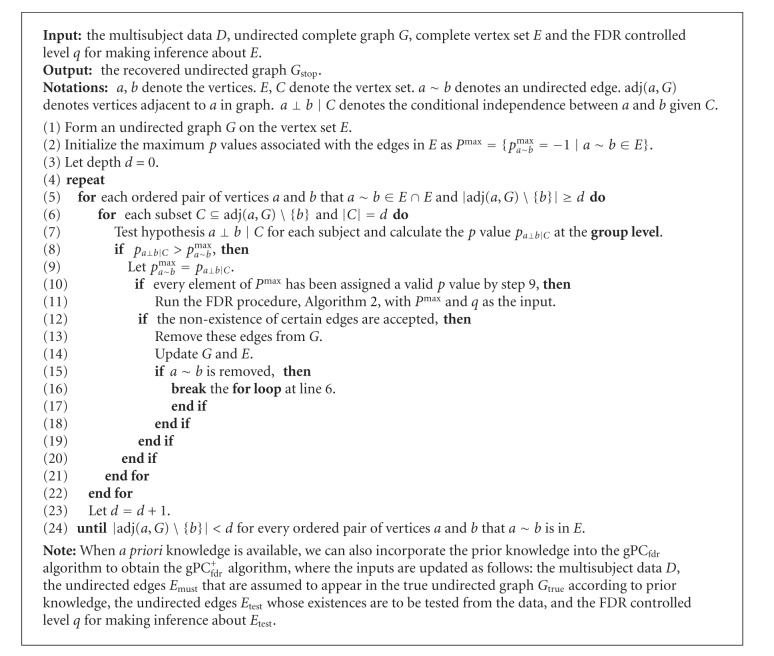
The gPC_fdr_ algorithm.

**Table 1:** Brain regions of interest (ROIs).

Full name of brain region	Abbreviation
Left/right lateral cerebellar hemispheres	lCER, rCER
Left/right globus pallidus	lGLP, rGLP
Left/right putamen	lPUT, rPUT
Left/right supplementary motor cortex	lSMA, rSMA
Left/right thalamus	lTHA, rTHA
Left/right primary motor cortex	lM1, rM1

“l” or “r” in the abbreviations stands for “Left” or “Right,” respectively.

## Appendices

### A. Proof of Theorems

To assist the reading, we list below notations frequently used in the proof.

V: all the nodes in a graph,
Gtrue: the skeleton of the true underlying directed acyclic graph (DAG),
𝒜a~b: the event that edge *a* ~ *b* is in the graph recovered by the PC_fdr_^+^ algorithm,
𝒜Etrue′: *𝒜*_*E*_true_′_ = ⋂_*a*~*b*∈*E*_true_′_ *𝒜*_*a*~*b*_, the joint event that all the edges in *E*_true_′, the true edges in *E*_test_, are recovered by the PC_fdr_^+^ algorithm,
pa~b: the value of *p*_*a*~*b*_^max^ when the PC_fdr_^+^ algorithm stops,
Ca~b*: a certain vertex set that *d*-separates *a* and *b* in the true DAG and that is also a subset of either adj(*a*, *G*_true_)∖{*b*} or adj(*b*, *G*_true_)∖{*a*}, according to Proposition 1. *C*_*a*~*b*_* is defined only for vertex pairs that are not adjacent in *G*_true_,
pa~b*: the *p* value of testing *X*_*a*_⊥*X*_*b*_ | *X*_*C*_*a*~*b*_*_. The conditional independence relationship may not be really tested during the process of the PC_fdr_^+^ algorithm, but *p*_*a*~*b*_* can still denote the value as if the conditional independence relationship was tested,
H*: the value in (*⋆*) in Algorithm 2 that is either *H* or *H*(1 + 1/2,…, +1/*H*), depending on the assumption of the dependency of the *p* values.

Lemma A.1 If *𝒜*_*i*_(*m*),…, *𝒜*_*K*_(*m*) are a finite number of events whose probabilities each approach 1 as *m* approaches infinity

(A.1) $$\begin{array}{c} \underset{m\to \infty }{\lim}P\left({𝒜}_{i}\left(m\right)\right)=1, \end{array}$$

then the probability of the joint of all these events approaches 1 as *m* approaches infinity:

(A.2) $$\begin{array}{c} \underset{m\to \infty }{\lim}P\left(\overset{K}{\underset{i=1}{⋂}}{𝒜}_{i}\left(m\right)\right)=1. \end{array}$$

For the proof of this lemma, please refer to Li and Wang's [11] work.

Lemma A.2 If there are *F* (*F* ≥ 1) false hypotheses among *H* tested hypotheses and the *p* values of the all the false hypotheses are smaller than or equal to (*F*/*H**)*q*, where *H** is either *H* or *H*(1 + 1/2,…, +1/*H*) depending on the assumption of the dependency of the *p* values, then all the *F* false hypotheses will be rejected by the FDR procedure, Algorithm 2.

For the proof of this lemma, please refer to Li and Wang's [11] work.

Proof of Theorem 2
If there is not any true edge in *E*_true_′, that is, *E*_true_′ = *∅*, then the proof is trivially *E*_true_′ = *∅*⊆*E*′. In the following part of the proof, we assume *E*_true_′ ≠ *∅*. For the PC_fdr_^+^ algorithm and its heuristic modification, whenever the FDR procedure, Algorithm 2, is invoked, *p*_*a*~*b*_^max^ is always less than max_*C*∈*V*∖{*a*,*b*}_{*p*_*a*⊥*b*∣*C*_}, and the number of *p* values input to the FDR algorithm is always not more than |*E*_test_|. Thus, according to Lemma A.2, if

(A.3) $$\begin{array}{c} \underset{a\sim b\in E_{\text{true}}^{\prime }}{\max}\left\{\underset{C\in V\setminus \{a,b\}}{\max}\left\{p_{a⊥b\;|\;C}\right\}\right\}\leq \frac{\left|E_{\text{true}}^{\prime }\right|}{\left|E_{\text{test}}\right|\sum_{i=1}^{\left|E_{\text{test}}\right|}\left(1/i\right)}q, \end{array}$$

then all the true connections will be recovered by the PC_fdr_^+^ algorithm and its heuristic modification. Let *𝒜*_*a*⊥*b*∣*C*_′ denote the event

(A.4) $$\begin{array}{c} p_{a⊥b\;|\;C}\leq \frac{\left|E_{\text{true}}^{\prime }\right|}{\left|E_{\text{test}}\right|\sum_{i=1}^{\left|E_{\text{test}}\right|}\left(1/i\right)}q, \end{array}$$

*𝒜*_*E*_true_′_′ denote the event of (A.3), and *𝒜*_*E*_true_′_ denote the event that all the true connections in *E*_test_ are recovered by the PC_fdr_^+^ algorithm and its heuristic modification.

∵*𝒜*_*E*_true_′_′ is a sufficient condition for *𝒜*_*E*_true_′_, according to Lemma A.2.
∴*𝒜*_*E*_true_′_⊇*𝒜*_*E*_true_′_′.
∴*P*(*𝒜*_*E*_true_′_) ≥ *P*(*𝒜*_*E*_true_′_′).
∵*𝒜*_*E*_true_′_′ is the joint of a limited number of events as
  (A.5) $$\begin{array}{c} {𝒜}_{E_{\text{true}}^{\prime }}^{\prime }=\underset{a\sim b\in E_{\text{true}}^{\prime }}{⋂}‍\;\underset{C\subseteq V\setminus \left\{a,b\right\}}{⋂}{𝒜}_{a⊥b\;|\;C}^{\prime }, \end{array}$$
and lim_*m*→*∞*_ *P*(*𝒜*_*a*⊥*b*∣*C*_′) = 1 according to Assumption (A3).
∴ According to Lemma A.1, lim_*m*→*∞*_ *P*(*𝒜*_*E*_true_′_′) = 1.
∴1 ≥ lim_*m*→*∞*_ *P*(*𝒜*_*E*_true_′_) ≥ lim_*m*→*∞*_ *P*(*𝒜*_*E*_true_′_′) = 1.
∴lim_*m*→*∞*_ *P*(*𝒜*_*E*_true_′_) = 1.

Lemma A.3 Given any FDR level *q* > 0, if the *p* value vector *P* = [*p*_1_,…, *p*_*H*_] input to Algorithm 2 is replaced with *P*′ = [*p*_1_′,…, *p*_*H*_′], such that (1) for the those hypotheses that are rejected when *P* is the input, *p*_*i*_′ is equal to or less than *p*_*i*_, and (2) for all the other hypotheses, *p*_*i*_′ can be any value between 0 and 1, then the set of rejected hypotheses when *P*′ is the input is a superset of those rejected when *P* is the input.

For the proof of this lemma, please refer to Li and Wang's [11] work.

Corollary A.4 Given any FDR level *q* > 0, if the *p* value vector *P* = [*p*_1_,…, *p*_*H*_] input to Algorithm 2 is replaced with *P*′ = [*p*_1_′,…, *p*_*H*_′] such that *p*_*i*_′ ≤ *p*_*i*_ for all *i* = 1,…, *H*, then the set of rejected hypotheses when *P*′ is the input is a superset of those rejected when *P* is the input.

The corollary can be easily derived from Lemma A.3.

Proof of Theorem 3
Let *P*_stop_ = {*p*_*a*~*b*_} denote the value of *P* ^max^ when the PC_fdr_-skeleton algorithm stops.

∵ The FDR procedure is invoked whenever *P* ^max^ is updated, and *P* ^max^ keeps increasing as the algorithm progresses.
∴ According to Corollary A.4, *E*_stop_′ is the same as the edges recovered by directly applying the FDR procedure to *P*_stop_.

The theorem is proved through comparing the result of the PC_fdr_^+^ algorithm with that of applying the FDR procedure to a virtual *p* value set constructed from *P*_stop_. The virtual *p* value set *P** is defined as follows. For a vertex pair *a* ~ *b* that is not adjacent in *G*_true_, let *C*_*a*~*b*_* denote a certain vertex set that *d*-separates *a* and *b* in the true graph and that is also a subset of either adj(*a*, *G*_true_)∖{*b*} or adj(*b*, *G*_true_)∖{*a*}. Let us define *P** = {*p*_*a*~*b*_* | *a* ~ *b* ∈ *E*_test_} as

(A.6) $$\begin{array}{c} p_{a\sim b}^{*}=\{\begin{array}{cc} p_{a⊥b|C_{a\sim b}^{*}}: & a\sim b\notin E_{\text{true}}^{\prime },\\ p_{a\sim b}: & a\sim b\in E_{\text{true}}^{\prime }. \end{array} \end{array}$$

Though *p*_*a*⊥*b*|*C*_*a*~*b*_*_ may not be actually calculated during the process of the algorithm, *p*_*a*⊥*b*|*C*_*a*~*b*_*_ still can denote the value as if it was calculated. Let us design a virtual algorithm, called *Algorithm**, that infers true edges in *E*_test_ by just applying the FDR procedure to *P**, and let *E** denote the edges in *E*_test_ claimed to be true by this virtual algorithm. This algorithm is virtual and impracticable because the calculation of *P** depends on the unknown *E*_true_′, but this algorithm exists because *E*_true_′ exists. For any vertex pair *a* and *b* that is not adjacent in *G*_true_, we have the following.

∵*X*_*a*_ and *X*_*b*_ are conditional independent given *X*_*C*_*a*~*b*_*_.
∴*p*_*a*⊥*b*|*C*_*a*~*b*_*_ follows the uniform distribution on [0, 1].
∴ The FDR of *Algorithm** is under *q*.

When all the true edges in the test set are recovered by the PC_fdr_^+^ algorithm, that is, *E*_true_′⊆*E*_stop_′, all the edges in *G*_true_ are included in *E*_stop_ due to Assumption (A4). In this case, the conditional independence between *X*_*a*_ and *X*_*b*_ given *X*_*C*_*a*~*b*_*_ is tested for all the falsely recovered edges *a* ~ *b* ∈ *E*_stop_′∖*E*_true_′, because for these edges, subsets of adj(*a*, *G*_true_)∖{*b*} and subsets of adj(*a*, *G*_true_)∖{*b*} have been exhaustively searched and *C*_*a*~*b*_* is one of them. Therefore, *p*_*a*~*b*_ ≥ *p*_*a*~*b*_* for all *a* ~ *b* ∈ *E*_stop_′ when event *𝒜*_*E*_true_′_ happens. Consequently, according to Lemma A.3,

(A.7) if  event  𝒜Etrue′  happens,Estop′⊆E∗.

Let *q*(*E*′) denote the *realized* FDR of reporting *E*′ as the set of true edges in *E*_test_:

(A.8) q(E′)={|E′∖Etrue′||E′|:E′≠∅,0:E′=∅.

The FDRs of the PC_fdr_^+^ algorithm and *Algorithm** are *E*[*q*(*E*_stop_′)] and *E*[*q*(*E**)], respectively. Here *E*[*x*] means the expected value of *x*.

$∵E[q(E_{\text{stop}}^{\prime })]=E[q(E_{\text{stop}}^{\prime })|{𝒜}_{E_{\text{true}}^{\prime }}]P({𝒜}_{E_{\text{true}}^{\prime }})+E[q(E_{\text{stop}}^{\prime })|{\overset{-}{𝒜}}_{E_{\text{true}}^{\prime }}]P({\overset{-}{𝒜}}_{E_{\text{true}}^{\prime }})\leq Q\;+\;P({\overset{-}{𝒜}}_{E_{\text{true}}^{\prime }})$, where *Q* = *E*[*q*(*E*_stop_′) | *𝒜*_*E*_true_′_]*P*(*𝒜*_*E*_true_′_).
∴${\mathrm{limsup}}_{m\to \infty }E[q(E_{\text{stop}}^{\prime })]\leq {\mathrm{limsup}}_{m\to \infty }Q+{\mathrm{limsup}}_{m\to \infty }P({\overset{-}{𝒜}}_{E_{\text{true}}^{\prime }})$.
∵lim_*m*→*∞*_ *P*(*𝒜*_*E*_true_′_) = 1, according to Theorem 2.
∴${\mathrm{limsup}}_{m\to \infty }P({\overset{-}{𝒜}}_{E_{\text{true}}^{\prime }})=\lim_{m\to \infty }P({\overset{-}{𝒜}}_{E_{\text{true}}^{\prime }})=0$.
∴limsup_*m*→*∞*_ *E*[*q*(*E*_stop_′)] ≤ limsup_*m*→*∞*_ *Q*.
∵*Q* ≤ *E*[*q*(*E*_stop_′)].
∴limsup_*m*→*∞*_ *Q* ≤ limsup_*m*→*∞*_ *E*[*q*(*E*_stop_′)].
∴limsup_*m*→*∞*_ *E*[*q*(*E*_stop_′)] = limsup_*m*→*∞*_ *Q* = limsup_*m*→*∞*_ *E*[*q*(*E*_stop_′) | *𝒜*_*E*_true_′_]*P*(*𝒜*_*E*_true_′_).

Similarly, limsup_*m*→*∞*_ *E*[*q*(*E**)] = limsup_*m*→*∞*_ *E*[*q*(*E**) | *𝒜*_*E*_true_′_]*P*(*𝒜*_*E*_true_′_).

∵ Event *𝒜*_*E*_true_′_ implies *E*_true_′⊆*E*_stop_′⊆*E**.
∴ Given event *𝒜*_*E*_true_′_,
  (A.9) q(Estop′)=|Estop′|−|Etrue′||Estop′|=1−|Etrue′||Estop′|≤1−|Etrue′||E∗|=|E∗|−|Etrue′||E∗|=q(E∗).
∴limsup_*m*→*∞*_ *E*[*q*(*E*_stop_′) | *𝒜*_*E*_true_′_]*P*(*𝒜*_*E*_true_′_) ≤ limsup_*m*→*∞*_ *E*[*q*(*E**) | *𝒜*_*E*_true_′_]*P*(*𝒜*_*E*_true_′_).
∴limsup_*m*→*∞*_ *E*[*q*(*E*_stop_′)] ≤ limsup_*m*→*∞*_ *E*[*q*(*E**)].
∵*Algorithm** controls the FDR under *q*.
∴*E*[*q*(*E**)] ≤ *q*.
∴limsup_*m*→*∞*_ *E*[*q*(*E**)] ≤ *q*.
∴limsup_*m*→*∞*_ *E*[*q*(*E*_stop_′)] ≤ *q*.

### B. Computational Complexity

The PC_fdr_^+^ algorithm spends most of its computation on performing statistical tests of conditional independence at step 7 and controlling the FDR at step 11. If the algorithm stops at the depth *d* = *d*_max_, then the number of conditional independence tests required is bounded by

(B.1) T=2|Etest|∑d=0dmaxCΔ−1d,

where |*E*_test_| is the number of edges to test, Δ is the maximum degree of graph *G*_init_ (the graph formed at step 1 of the PC_fdr_^+^ algorithm) whose edges are *E*_must_∩*E*_test_, and *C*_Δ−1_^*d*^ is the number of combinations of choosing *d* unordered and distinct elements from Δ − 1 elements. In the worst case that *d*_max_ = Δ − 1, the complexity is bounded by 2 | *E*_test_ | 2^Δ−1^ = |*E*_test_ | 2^Δ^. The bound usually is very loose, because it assumes that no edge has been removed until *d* = *d*_max_. In real-world applications, the algorithm is very fast for sparse networks.

The computational complexity of the FDR procedure, Algorithm 2, invoked at step 11 of the PC_fdr_^+^ algorithm, in general is *O*(*H*log(*H*) + *H*) = *O*(*H*log(*H*)) where *H* = |*E*_test_| is the number of input *p* values. The main complexity *H*log(*H*) is at the sorting (step 1). However, if it is recorded the sorted *P* ^max^ of the previous invocation of the FDR procedure, then the complexity of keeping the updated *P* ^max^ sorted is only *O*(*H*). With this optimization, the complexity of the FDR-control procedure is *O*(*H*log(*H*)) at its first operation and is *O*(*H*) later. The FDR procedure is invoked only when *p*_*a*⊥*b*∣*C*_ > *p*_*a*~*b*_^max^. In the worst case that *p*_*a*⊥*b*∣*C*_ is always larger than *p*_*a*~*b*_^max^, the complexity of the computation spent on the FDR control in total is bounded by *O*(|*E*_test_ | log(|*E*_test_|) + *T* | *E*_test_|) where *T* is the number of performed conditional independence tests. This is a very loose bound because it is rare that *p*_*a*⊥*b*∣*C*_ is always larger than *p*_*a*~*b*_^max^.

## References

[1] Smith SM, Miller KL, Salimi-Khorshidi G (2011). Network modelling methods for FMRI. *NeuroImage*.

[2] Friston KJ (2011). Functional and effiective connectivity: a review. *Brain Connectivity*.

[3] McIntosh AR, Gonzalez-Lima F (1994). Structural equation modeling and its application to network analysis in functional brain imaging. *Human Brain Mapping*.

[4] Friston KJ, Harrison L, Penny W (2003). Dynamic causal modelling. *NeuroImage*.

[5] Li J, Wang ZJ, Eng JJ, McKeown MJ (2008). Bayesian network modeling for discovering “dependent synergies” among muscles in reaching movements. *IEEE Transactions on Biomedical Engineering*.

[6] Benjamini Y, Yekutieli D (2001). The control of the false discovery rate in multiple testing under dependency. *Annals of Statistics*.

[7] Storey JD (2002). A direct approach to false discovery rates. *Journal of the Royal Statistical Society B*.

[8] Listgarten J, Heckerman D Determining the number of non-spuriousarcs in a learned DAG model: investigation of a Bayesian and a frequentist approach.

[9] Tsamardinos I, Brown LE Bounding the false discovery rate in local bayesian network learning.

[10] Spirtes P, Glymour C, Scheines R (2001). *Causation, Prediction, and Search*.

[11] Li J, Wang ZJ (2009). Controlling the false discovery rate of theassociation/causality structure learned with the pc algorithm. *Journal of Machine Learning Research*.

[12] Worsley KJ, Liao CH, Aston J (2002). A general statistical analysis for fMRI data. *NeuroImage*.

[13] Friston KJ, Stephan KE, Lund TE, Morcom A, Kiebel S (2005). Mixed-effects and fMRI studies. *NeuroImage*.

[14] Stephan KE, Penny WD, Daunizeau J, Moran RJ, Friston KJ (2009). Bayesian model selection for group studies. *NeuroImage*.

[15] Varoquaux G, Gramfort A, Poline JB, Thirion B (2010). Brain covariance selection: better individual functional connectivity models using population prior. *Advances in Neural Information Processing Systems*.

[16] Ramsey JD, Hanson SJ, Glymour C (2011). Multi-subject search correctly identifies causal connections and most causal directions in the DCM models of the Smith et al. simulation study. *NeuroImage*.

[17] Lauritzen SL (1996). *Graphical Models*.

[18] Neyman J, Pearson ES (1928). On the use and interpretation of certaintest criteria for purposes of statistical inference: part I. *Biometrika*.

[19] Fisher RA (1915). Frequency distribution of the values of the correlation 40 coefficients in samples from an indefinitely large population. *Biometrika*.

[20] Palmer SJ, Ng B, Abugharbieh R, Eigenraam L, McKeown MJ (2009). Motor reserve and novel area recruitment: amplitude and spatial characteristics of compensation in Parkinson’s disease. *European Journal of Neuroscience*.

